# Parameters and Measures in Assessment of Motor Learning in Neurorehabilitation; A Systematic Review of the Literature

**DOI:** 10.3389/fnhum.2017.00082

**Published:** 2017-02-24

**Authors:** Nataliya Shishov, Itshak Melzer, Simona Bar-Haim

**Affiliations:** Department of Physical Therapy, Recanati School for Community Health Professions, Faculty of Health Sciences, Ben-Gurion University of the NegevBeer-Sheva, Israel

**Keywords:** neurorehabilitation, stroke, cerebral palsy, motor learning, assessment, systematic review

## Abstract

Upper limb function, essential for daily life, is often impaired in individuals after stroke and cerebral palsy (CP). For an improved upper limb function, learning should occur, and therefore training with motor learning principles is included in many rehabilitation interventions. Despite accurate measurement being an important aspect for examination and optimization of treatment outcomes, there are no standard algorithms for outcome measures selection. Moreover, the ability of the chosen measures to identify learning is not well established. We aimed to review and categorize the parameters and measures utilized for identification of motor learning in stroke and CP populations. PubMed, Pedro, and Web of Science databases were systematically searched between January 2000 and March 2016 for studies assessing a form of motor learning following upper extremity training using motor control measures. Thirty-two studies in persons after stroke and 10 studies in CP of any methodological quality were included. Identified outcome measures were sorted into two categories, “parameters,” defined as identifying a form of learning, and “measures,” as tools measuring the parameter. Review's results were organized as a narrative synthesis focusing on the outcome measures. The included studies were heterogeneous in their study designs, parameters and measures. Parameters included adaptation (*n* = 6), anticipatory control (*n* = 2), after-effects (*n* = 3), de-adaptation (*n* = 4), performance (*n* = 24), acquisition (*n* = 8), retention (*n* = 8), and transfer (*n* = 14). Despite motor learning theory's emphasis on long-lasting changes and generalization, the majority of studies did not assess the retention and transfer parameters. Underlying measures included kinematic analyses in terms of speed, geometry or both (*n* = 39), dynamic metrics, measures of accuracy, consistency, and coordination. There is no exclusivity of measures to a specific parameter. Many factors affect task performance and the ability to measure it—necessitating the use of several metrics to examine different features of movement and learning. Motor learning measures' applicability to clinical setting can benefit from a treatment-focused approach, currently lacking. The complexity of motor learning results in various metrics, utilized to assess its occurrence, making it difficult to synthesize findings across studies. Further research is desirable for development of an outcome measures selection algorithm, while considering the quality of such measurements.

## Introduction

Neurological disorders affect a significant amount of people worldwide. Two common disorders are stroke and cerebral palsy (CP). Stroke occurs due to interruption of the blood supply to the brain or as a result of ischemia or bleeding (WHO, 2006), and has a prevalence of ~795,000 new or recurrent events in the United States each year (Lloyd-Jones et al., 2010). CP is the most common neurodevelopmental motor disorder in children, which begins in early childhood and persists throughout lifespan (Bax et al., 2005), with a prevalence of 2–2.5 per 1,000 live births (Himmelmann, 2013). A common problem experienced by these populations is impaired upper extremity function. About 70% of stroke survivors lose motor skills of the paretic arm and hand (Lloyd-Jones et al., 2010). Even mild impairment results in significant daily function limitations and has a negative impact on the quality of life (Lai et al., 2002; Nichols-Larsen et al., 2005). Thirty-five percent of children with CP are diagnosed with hemiplegia, with their upper limb usually more affected than the lower extremity (Wiklund and Uvebrant, 1991). Regaining optimal upper extremity function is essential for participation in daily life, and for this reason, is one of the goals of neurorehabilitation.

During rehabilitation, “a process of relearning how to move to carry out their needs successfully” (Carr and Shepherd, 1987), patients improve their activity by either development of compensatory strategies (i.e., generation of the motor task with alternative movement patterns) or by reacquisition of the pre-lesion patterns, defined as recovery (Levin et al., 2009). Despite the difference in the underlying neuronal mechanisms of compensation and recovery (Tanaka et al., 2011), they both require learning (Kitago and Krakauer, 2013). Therefore, a great amount of therapeutic interventions apply motor learning principles, assuming these principles can enhance motor recovery and that permanent improvements in motor function can be achieved by training (Kitago and Krakauer, 2013). Motor learning was defined as “a set of internal processes associated with practice or experience, leading to a relatively permanent change in the capability for movement” (Schmidt, 1988). As these internal neural and cognitive processes cannot be directly observed nor measured at the behavioral level, motor learning can be estimated only by observing the performance (Cahill et al., 2001; Schmidt and Wrisberg, 2008).

The questions whether neurological patients are capable of learning and whether they have specific motor learning deficits are difficult to definitively answer due to the variety of motor tasks that rely on different learning processes, all associated with various functional and anatomical brain structures (Krakauer and Mazzoni, 2011; Kitago and Krakauer, 2013). Moreover, the heterogeneity of patients, some having additional impairments masking their learning abilities, can make it difficult to demonstrate learning abnormalities (Krakauer, 2006; Kitago and Krakauer, 2013).

Several systematic reviews looked into measurable parameters in the rehabilitation process. Huang and Krakauer (2009) reviewed studies that explored the change in rehabilitation outcome as a function of different aspects of the intervention, such as amount, type, timing, and intensity of practice, and their effects on post stroke rehabilitation. Because motor learning is compiled from various processes (Krakauer and Mazzoni, 2011), diverse parameters are used to assess different learning types and aspects. For evaluation of an intervention's efficacy it is important to choose the appropriate measure (Huang and Krakauer, 2009). Huang and Krakauer (2009) distinguished between the adaptation and motor skill learning processes, placing the learning at a higher level of the motor control hierarchy. Also, various clinical outcome measures of functional performance [e.g., the Action Research Arm Test (ARAT; Carroll, 1965; Lyle, 1981), the Wolf Motor Function Test (WMFT; Wolf et al., 1989), Functional Independence Measure (FIM; Hamilton et al., 1994) etc.] were compared to a measurement of impairment [the Fugl-Meyer Motor Assessment (FMA; Fugl-Meyer et al., 1975; Gladstone et al., 2002)] (Huang and Krakauer, 2009). Sivan et al. (2011) identified outcome measures utilized in robot-assisted exercise therapy in stroke patients. Measures were clustered based on which domain within the International Classification of Functioning, Disability and Health (ICF) they evaluate. From the ICF framework, the patient characteristics and the reliability and validity of measures, an algorithm for selection of outcome measures was suggested. Kinematic measures, FIM, FMA, and the WMFT were identified as suitable for use in robot trainings, each for a different ICF domain, severity of impairment and time since stroke (Sivan et al., 2011). Only stroke patients undergoing a robot therapy intervention were included. The time of performance evaluation throughout the training and the relation of these measures to motor learning were not focused on. On the other hand, Kantak and Winstein (2012) highlighted that performance during acquisition might be transient and influenced by independent factors. They suggested that implementation of retention or transfer tests, which measure lasting improvements of motor execution of a skill, are essential in order to infer learning (Kantak and Winstein, 2012). Overall, these reviews focused little or not at all on application of common measurements to understanding the motor learning process.

Currently, there are no standard procedures regarding the choice of outcome measures (Huang and Krakauer, 2009). Inaccurate deduction of learning, caused by inadequate metric selection, might for example suggest a failure of training, when in fact inaccurate choice of measure is at fault. Moreover, reliable assessment and understanding of patients' motor learning process may reveal the impaired component within the process, and therefore facilitate the development and selection of an adequate and specific treatment to enhance recovery (Kitago and Krakauer, 2013). The assessments and measures of motor learning should demonstrate sensitivity to relevant change and remain invariable when there is no change in function.

Our main goal was to review the different parameters and underlying measures used in available studies to assess and measure the occurrence of motor learning following an intervention. We aimed to categorize the different parameters depending on the type and process of learning they evaluate, and to present the characteristics of the different parameters. Understanding the timing, purpose, advantages, and disadvantages of parameters and measures, will help both researchers and clinicians to better design studies, evaluate patients and treatment efficiency, compare interventions and better understand the underlying mechanisms of recovery. To the best of our knowledge no such comprehensive collection of information has previously been done.

## Methods

### Search strategy

PubMed, Pedro, and Web of Science databases were searched for studies published between January 2000 and March 2016. For PubMed and Web of Science databases the following key words were searched: (1) learning OR motor learning OR motor control AND (2) stroke OR cerebral palsy AND (3) parameter OR measure OR assessment OR adaptation OR acquisition OR retention OR transfer AND (4) upper limb OR upper extremity OR arm OR hand AND (5) rehabilitation OR treatment OR training. Due to the considerable number of key words, modification of the search strategy was made for the Pedro database, to match the database requirements. The following searches were performed: (1) cerebral palsy AND learning AND hand (2) cerebral palsy AND motor control and hand (3) stroke AND learning AND hand (4) stroke AND motor control AND hand. The key word “hand” was chosen to represent the upper limb studies, as from overview of the retrieved records, it included results of most studies performed on the upper extremity.

### Study selection

To be included in the review, a study had to: (1) involve either stroke survivors or persons with CP, (2) assess process or type of learning after an intervention with motor learning principles, (3) be applicable for rehabilitation, (4) use outcome measures relevant for motor control, (5) focus on the upper extremity, (6) be written in or translated to English. Studies were excluded from the review if the study: (1) did not focus on human subjects, (2) used only clinical outcome measures, (3) included an intervention of virtual reality, transcranial magnetic stimulation or of electric muscle stimulation.

Titles and abstracts of retrieved records were merged into one database on the reference management software, and duplicates were removed.

### Quality assessment

Titles and potential abstracts were screened independently by two researchers (First and last authors). Titles that contained any of the exclusion criteria were excluded based on title only. Relevant full text articles and full texts of abstracts that were inconclusive regarding their relevancy were assessed, and studies that did not correspond with the inclusion criteria were excluded. Fitting articles were also extracted from reviews relevant to the topic and from full text article references. Data regarding studies' designs was extracted. All study designs of any methodological quality were included. Due to our objective to perform a comprehensive data collection of the various parameters and measures, we did not factor the strength of experimental evidence provided by the studies. In addition to studies that examined the efficacy of an intervention, we included studies that explored the feasibility of tools, hypotheses regarding mechanisms of learning and recovery and the implementation of mathematical models. In such studies, assessment of the methodological quality would yield no benefit, due to their different objectives. A narrative synthesis of the literature was performed.

### Data collection and synthesis

First, all included articles were reviewed. Data was extracted and compiled from each research on: (1) studies' characteristics and methodology, (2) the grouping method, (3) type of intervention, (4) study protocol (5) outcome measures. We further gathered information regarding the characteristics of the outcome measures utilized to assess learning. Parameters utilized to infer learning were clustered according to the motor learning principle or form of learning that they assessed. Underlying measures for each parameter were defined as the measures used to quantify the parameter.

## Results

**Figure 1:**
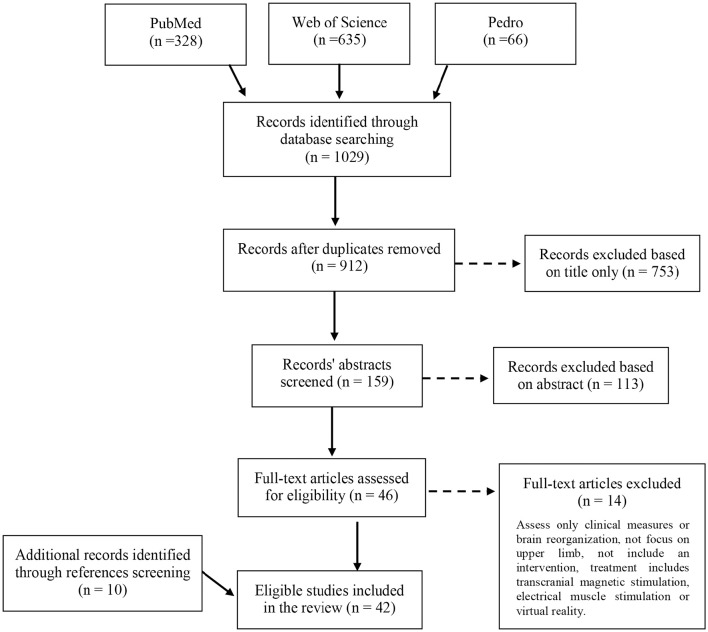
**Flow diagram of the studies' selection process for the review**.

The database search retrieved 1,029 records. After removal of duplicates and eligibility assessment of the remaining articles, 32 studies were included in the review. Ten additional studies were identified by scanning the reference lists of relevant full test articles and of reviews relevant to the topic. In total, 42 studies were included in the review (Figure 1).

Table 1 presents the study characteristics and methodological information of the 42 reviewed studies. Thirty-two of the studies addressed stroke patients, and 10 examined children or adolescents with CP. Our decision to focus on stroke and CP patients was due to their relatively high prevalence (Bax et al., 2005; Lloyd-Jones et al., 2010; Himmelmann, 2013). Also, they are a topic of considerable research because of their commonly affected motor skills of the upper extremity (Lai et al., 2002; Nichols-Larsen et al., 2005; Lloyd-Jones et al., 2010). Twenty-one studies trained and assessed learning of the paretic hand, seven studies of the less affected hand, and in 14 of the studies both hands were either trained, evaluated or both. In 16 of the 35 studies that assessed the motor learning by examining only the affected or both hands, the affected arm was reported to be supported against gravity, in the remaining studies the arm was not supported. Additionally, 17 studies reported minimizing compensatory strategies of the upper limb or trunk (by a belt, harness etc.). Only two studies (Cirstea and Levin, 2007; Massie et al., 2009) reported measurement of the compensations as part of the study protocol (Table 1).

**Table 1:** **Description of included studies and their characteristics**.

**Study**	**Diagnosis of intervention group and time since diagnosis**	**Intervention**	**Intervention group**	**Control group**	**Gender**	**Age in years**	**Protocol**	**Trained and assessed arm Gravity support for paretic arm Compensatory movements**
Aluru et al., 2014	Chronic stroke (>6 months)	Bimanual wrist extension task with auditory conditions	*N* = 20	No control	12M, 8F	Range 28–87	1st visit: clinical evaluations and matching of metronome speed for a positive mood-state. 2nd visit: two bimanual and one unimanual trials of: wrist flexion-extension movements with 4 auditory conditions: without cuing, positive sounds, self-selected positive mood state, and individual comfortable pace	Both arms Restricted compensatory movements of arm and forearm
Boyd and Winstein, 2001	Chronic unilateral sensimotor cortical (SMC) stroke (> 6 months)	Implicit motor sequence learning task with and without explicit information (EI) of the sequence	*N* = 12	No control	9M, 3F	Range 31–81	Serial reaction time task (SRTT) practice for 1 or 3 days, 24 trials each day	Less-affected
Boyd and Winstein, 2004	Chronic basal ganglia (BG) stroke (> 6 months)	Implicit motor sequence learning task with and without EI	*N* = 5 EI, *N* = 5 no-EI	Age matched Healthy Controls (HC) *N* = 5 EI *N* = 5 no-EI	BG EI: 4M, 1F BG no-EI: 3M, 2F HC EI: 1M, 4F HC no-EI: 2M, 3F	51 ± 9.8 58.2 ± 14.6 55.4 ± 11 57.4 ± 16.1	Continuous tracking (CT) task practice for 3 days, 50 trails each day	Less-affected
Boyd and Winstein, 2006	Chronic SMC chronic BG (> 6 months)	Implicit motor sequence learning task with and without EI	*N* = 10 BG *N =* 10 SMC	*N =* 10 Age matched HC	BG EI: 4M, 1F BG no-EI: 3M, 2F SMC EI: 2M, 3F SMC no-EI: 4M, 1F HC EI: 1M, 4F HC no-EI: 2M, 3F	51 ± 9.8 58.2 ± 14.6 59 ± 10.5 58.6 ± 19.2 55.4 ± 11 57.4 ± 16.1	SRTT and CT task practice for 3 days	Less-affected
Bourke et al., 2015	Unilateral sub-acute stroke (mean 27.5 days)	Postural perturbation task on a bilateral exoskeleton device	*N =* 38	*N =* 74 age and hand dominance matched HC	Stroke: 21M, 17F HC: 42M, 32F	Mean stroke: 63.5 HC: 62	Postural flexion/extension elbow perturbation task practice for 9 blocks, 8 trials in each block	Both arms Support against gravity
Burtner et al., 2014	Spastic hemiplegic CP	Learning of a discrete coordinated movement with 62 or 100% feedback frequencies	*N =* 19	*N =* 20 HC	CP: 9M, 19F HC: 12M, 8F	11.7 ± 2.4 10.8 ± 2	Replication of elbow extension-flexion reversal movement practice for 1 day	Less-affected
Caimmi et al., 2008	Chronic stroke (22 ± 7 months)	CIMT	*N =* 8	*N =* 8 HC	Stroke: 4M, 4F HC: 5M, 3F	53 ± 8 50 ± 4	CIMT for 14 days, 12 h a day and 1 h of occupational therapy (weekends) + 1 h of physiotherapy (weekdays)	Practice: paretic Assessment: both Trial repetition if trunk compensatory movement occurred
Casadio and Sanguineti, 2012	Chronic stroke (range 12–76 months)	Robotic therapy	*N =* 9	No control	2M, 7F	Range 30–72	Reaching movements toward outer targets training with robot generated assistive force that was gradually reduced with improvement. 10 weeks, 1 session a week, 1 h each	Paretic
Chang et al., 2007	Chronic stroke (> 6 months)	Robotic therapy and traditional rehabilitation (TR)	*N =* 20 arm reaching was assessed only for *N =* 15	No control	12M, 8F	57.1 ± 14	Part 1 (30 min): symmetric push and pull bilateral movements with 10 or 20% of maximal isokinetic strength for 8 weeks, 24 session Part 2: (10 min) range of motion exercise, muscle tone normalization, compensatory activity of daily living training, postural control and gait correction training	Practice: both Assessment: paretic
Chen et al., 2012	Unilateral CP	Home environment CIMT (1) vs. TR (2)	(1) *N =* 24	(2) *N =* 23	(1) 12F, 12M (2) 12F, 11M	(1) 8.7 ± 1.9 (2) 8.8 ± 2	3.5–4 h a day, 2 days a week, for 4 weeks of: (1) functional tasks training for the more affected arm (2) neurodevelopmental treatment techniques	Training: (1) more affected (2) both Assessment: affected; Support against gravity Restriction of trunk compensation
Chen et al., 2014			(1) *N =* 23	(2) *N =* 22	(1) 12F, 11M (2) 12F, 10M	(1) 8.73 ± 1.9 (2) 8.66 ± 2		
Christopher and Johnson, 2014	Chronic stroke	Robotic therapy	*N =* 2	No control	1F, 1M	47, 71	Reaching and grasping tasks for 1 h a day, 3 days per week, for 4 weeks	Training: paretic Assessment: both
Colombo et al., 2008	Acute, subacute (1) and chronic (2) stroke (1) 2.1 ± 1.3; (2) 20.8 ± 12.6 months	Robotic therapy	(1) *N =* 9 (2) *N =* 13	No control	Not specified	(1) 57.4 ± 14.4 (2) 54.5 ± 12.5	Training of point-to-point reaching movements in the horizontal plane, 5 days a week for at least 3 weeks	Paretic; Support against gravity Restriction of trunk compensation
Cirstea and Levin, 2007	Chronic stroke (range 3–24 months)	Reaching movements with (1) knowledge of results (KR) vs. (2) knowledge of performance (KP) feedbacks	(1) *N =* 14 (2) *N =* 14	*N =* 5 HC only KR	(1) 10M, 4F (2) 7M, 7F HC: not specified	55.7 ± 15.4 59.1 ± 17.9 not specified	Training of point-to-point reaching movements to the contralateral workspace in the horizontal plane for 2 weeks	Paretic; No support against gravity Upper limb and trunk compensatory movements were permitted and measured
Dancause et al., 2002	Unilateral chronic stroke, mild to severe Hemiparesis (range 6 months—2.3 years)	Rapid elbow flexion movements with unexpected load	*N =* 10	*N =* 6 HC	Stroke: 7F, 3M HC: not specified	47.1 ± 13.4 23.6 ± 2	50^0^ movements toward a wide target with unexpected spring-like load in 30% of trials with visual feedback at the end of each trial	Paretic; Support against gravity
Dipietro et al., 2007	Chronic stroke (> 6 months)	Robotic therapy	*N =* 117	No control	Not specified	58.8 ± 1	18 sessions of point-to-point reaching movements practice for 6 weeks, 3 sessions per week	Paretic; Support against gravity Restriction of compensatory movements
Dipietro et al., 2009	Chronic stroke (range 10.7–54.7 months)		*N =* 47		31M, 16F	Mean 57.79		
Dipietro et al., 2012	Subacute (1) and chronic (2) unilateral focal stroke (1) 19.1 ± 1.2 days (2) 1150 ± 90 days		(1) *N =* 42 (2) *N =* 116		(1) 57% M (2) 63% M	(1) 61.3 ± 1.8 (2) 58.8 ± 1.2		
Durham et al., 2014	Stroke (<18 months)	Reach-to-grasp training with external focus (EF) vs. internal focus (IF) feedbacks	(1) EF → IF: *N =* 21 (2) IF → EF: *N =* 21		(1) 15M, 6F (2) 15M, 6F	(1) 59 ± 14 (2) 63 ± 13	Three types of reaching tasks: grasping a jar, transferring the hand to an object while opening and closing the hand and moving an object	Paretic; Trunk movement was not restricted
Geerdink et al., 2013	Unilateral spastic CP	(1) Modified CIMT and Bimanual training vs. (2) regular rehabilitation	(1) *N =* 28	(2) *N =* 22	(1) 14M, 14F (2) 8F, 14M	4.8 ± 1.3	(1) CIMT training for 6 weeks (54 h) followed by 2 weeks of Bimanual training (18h)	Practice: both Assessment: affected; No restriction against gravity
Gilliaux et al., 2015	CP	Conventional therapy with robot-assisted therapy (1) vs. conventional therapy (2)	(1) *N =* 8	(2) *N =* 8	Not specified	(1) 10.8 ± 4.6 (2) 11 ± 3.5	(1) Reaching movements toward motionless and dynamic targets, 3 sessions + 2 sessions of conventional therapy a week; (2) Neurodevelopmental therapy, 5 sessions a week, for 8 weeks	Paretic Support against gravity
Hemayattalab and Rostami, 2010	Unilateral CP	New motor skill learning task - throwing darts toward a target with different KR feedback frequencies	*N =* 8 0% *N =* 8 50% *N =* 8 100%	No control	Not specified	Range 7–15	One day training of 30 throws in 8 sessions	Affected No restriction against gravity
Hemayattalab et al., 2013	Spastic hemiplegic CP	New motor skill learning task – throwing beanbags toward a target	*N =* 20	No control	Not specified	11.6 ± 1.5	Training of 80 throws, subjects did not see the target and received verbal feedback at the end of each trial	Affected No restriction against gravity
Kitago et al., 2013	Chronic stroke (> 6 months)	CIMT	*N =* 10	No control	5F, 5M	Range 57–83	4 h a day for 2 weeks	Practice: paretic Assessment: both Compensatory strategies were reduced
Kitago et al., 2015	Chronic stroke (> 6 months)	Robotic therapy	*N =* 9	*N =* 14 HC	Stroke: 1F, 8M HC: 9F, 5M	60.8 ± 9.4	Goal directed exercises with a robotic assistive device (e.g. forward thrust; circle; reaching: horizontal, forward, star and zigzag patterns; mimicking; bringing a cup to mouth) for 3 weeks, 3 days a week, 2 sessions a day, 1.5 h each	Paretic Support against gravity Restriction of trunk compensation
Krebs et al., 2012	Hemiplegic CP (1) and traumatic brain injury (2) (> 6 months before enrolment)	Robotic therapy	(1) *N =* 12 (2) *N =* 1	No control	Not specified	Range 5–12	Point-to-point reaching movements toward 1 of 8 spaced targets practice for 8 weeks, 2 sessions per week	Affected No support against gravity Restriction of trunk movement
Masia et al., 2011	Hemiplegic CP	Robotic therapy	*N =* 7	*N =* 7 age matched HC	CP: 7M	Mean CP: 10.14 HC: 9	Practice of 640 (40 sets) center-out reaching movements toward 1 of 8 peripheral targets with applied force fields	Affected Support against gravity Restriction of wrist and trunk compensatory movements
Massie et al., 2009	Unilateral chronic stroke (> 9 months)	CIMT	*N =* 10	No control	3F, 7M	61 ± 14.7	6 h a day for 10 week days	Training: paretic Assessment: both No support against gravity Compensatory movements were permitted and measured
Mawase et al., 2011	Spastic diplegic CP	Grasp and lift task	*N =* 5	*N =* 5 age and gender matched HC	2F, 3M in each group	Mean CP: 26.8 HC: 25.4	Lifting a series of virtual objects with increasing weights (100–400 g)	Less affected
Molier et al., 2011	Chronic stroke (range 20–51 months)	Reaching training with resistance position feedback	*N =* 5	No control	2F, 3M	Range 50.8–68.7	Three reaching tasks (sliding hand over table, lifting and moving hand above table, lifting hand to a shelf) training for 30 min, three times a week for 6 weeks	Paretic; Restriction of trunk compensatory movements
Orrell et al., 2007	Chronic stroke (> 12 months)	Implicit motor sequence learning task	(1) *N =* 7	(2) *N =* 8 HC	(1) 5F, 2M	(1) 59.86 ± 10 (2) 47.4 ± 8	SRTT practice for 2 days	Less-affected
Patton et al., 2006	Chronic stroke (range 16–173 months)	Robotic therapy	*N =* 18	*N =* 4 HC	Not specified	Mean Stroke: 51 HC: 47	Reaching movements with applied forces practice	Paretic Support against gravity
Pohl et al., 2006	Subacute stroke (<45 days)	Implicit motor sequence learning task without EI	(1) *N =* 15 moderate stroke (2) *N =* 22 mild stroke	(3) *N =* 32 age matched HC	(1) 10F, 5M (2) 9F, 13M (3) 25F, 5M	(1) 74.4 ± 9.3 (2) 72.3 ± 8.5 (3) 76.4 ± 6.5	Practice of blocks with random and repeated sequence conditions (10 sets of 8-target sequence in each block) during which subjects were required to press 1 of 8 illuminated targets	Less affected
Raghavan et al., 2006	Subcortical stroke (> 3 months)	Grasping an object with thumb and index finger task	*N =* 8	*N =* 8 age matched HC	Not specified	Mean Stroke: 65.4 HC: 67.2	Following an auditory cue subjects grasped an object (that weighed 300/500 g randomly) with their thumb and index finger and lifted it to a 5 cm height over a table	Both arms Support against gravity by a table
Schaefer et al., 2013	Chronic stroke (52.7 ± 53.4 months)	Feeding task training	*N =* 11	No control	4F, 7M	58.9 ± 7.5	Spooning beans from one cup to another practice for 5 days	Paretic; No support against gravity
Scheidt and Stoeckmann, 2007	Chronic stroke (> 6 months)	Robotic therapy	*N =* 12	*N =* 6 HC	Stroke: 8M, 3F HC: 3F, 3M	Range 38–75	Reaching to a target with a peak hand speed of 0.5 ms, followed by graphical feedback of the hand speed	Both arms Support against gravity Restriction of trunk movement
Sterpi et al., 2012	Chronic stroke (> 6 months)	Robotic therapy	*N =* 18	No control	6F, 2M	Not specified	Training of a sequence of four point-to-point reaching movements (square path) for 3 weeks	Paretic Support against gravity Restriction of trunk movement
Senesac et al., 2010	Chronic stroke (5.5 ± 3.9 years)	Modified Bilateral arm training (BAT) with rhythmic auditory cueing	*N =* 14	No control	5F, 9M	64.4 ± 13.3	Subjects moved two handles back and forth for 2.25 h a day, 4 days a week, for 2 weeks. Trials included both in-phase and out-of-phase movements of handles	Both arms; Trunk movement was restricted during training but not during testing
Takahashi and Reinkensmeyer, 2003	Chronic stroke (> 3 months)	Robotic therapy	*N =* 13 Protocol with paretic hand	*N =* 13 Same subjects protocol with ipsilesional hand	4F, 9M	Range 34–89	Reaching movements on a lightweight robotic device with randomly applied force fields	Paretic No support against gravity
Thaut et al., 2002	Chronic stroke (11.4 ± 52 months)	Reaching movements with and without rhythmic temporal cuing	*N =* 21	No control	8F, 13M	52.7 ± 13.7	Subjects moved their arm back and forth for 30 sec between sensors, each subject completed a trial with and without cueing	Paretic
Wu et al., 2007	Stroke (range 3 weeks-37; mean 12.2 months)	(1) CIMT vs. (2) neurodevelopmental therapy	(1) *N =* 24	(2) *N =* 23	(1) 8F, 16M (2) 7F, 17M	(1) 53.9 ± 11.2 (2) 56.7 ± 12.9	(1) Training activities to the affected arm + 6 h a day restriction of the less affected arm (2) Functional tasks practice	Paretic Support against gravity by a table Restriction of trunk movement
Wu et al., 2011	Chronic stroke (mean 16.2 months)	(1) Distributed Constrained Induced Therapy vs. (2) BAT vs. (3) control treatment	(1) *N =* 22 (2) *N =* 22	(3) *N =* 22	(1) 7F, 15M (2) 4F, 18M (3) 6F, 16M	(1) 51.9 ± 11.9 (2) 52.2 ± 10.7 (3) 55.2 ± 2.5	2 h a day, 5 days a week for 3 weeks of: (1) unaffected hand restriction for 6 h daily and intensive functional tasks training for the affected hand; (2) symmetric or altering bilateral movements; (3) functional tasks practice for the affected (75%) and non-affected (25%) arms	Training: (1) paretic (2) both (3) both Assessment: both Support against gravity; Restriction of trunk movement

*The specified practiced and assessed hand is related to the motor learning measures, as in some of the studies additional clinical measures were examined (see Table 2). Data is presented by mean ± SD unless specified differently*.

*Abbreviations: M, Male; F, Female; EI, explicit information; HC, healthy controls; SRTT, Serial Reaction Time Task; CT, Continuous Tracking; TR, traditional rehabilitation; KR, knowledge of results; KP, knowledge of performance; CIMT, Constrained Induced Movement Therapy; BAT, Bilateral Arm Training*.

The parameters utilized to infer learning varied across the studies (Table 2) and included: adaptation, anticipatory control, after-effects, de-adaptation, performance, acquisition, reacquisition, retention, and transfer. Underlying variables used for measurement of the parameters included kinematic metrics in terms of timing, position, velocity, and acceleration, dynamic metrics of force generation, measures of accuracy, consistency, dexterity, and coordination. In all apart from three of the reviewed studies (Patton et al., 2006; Hemayattalab and Rostami, 2010; Sterpi et al., 2012), additional clinical measures were mentioned. In 17 of them clinical tests were performed only before the intervention, or before the beginning of the study for assessment of subjects' eligibility for participation (i.e., based on inclusion criteria). In 13 studies clinical measurements were taken before and after treatment, and in nine studies additional correlations were analyzed, or relationships examined, between the clinical and motor learning measures (Table 2). Apart from three studies (Hemayattalab and Rostami, 2010; Geerdink et al., 2013; Hemayattalab et al., 2013), all of the reviewed studies performed kinematic analysis of some sort for measurement of the parameter. In 18 of them both the velocity and accuracy components were evaluated, whereas in nine of them only the accuracy component of the movement was tested; 12 studies examined only the velocity component (Table 2). Six of the reviewed studies addressed measures of force generation (Raghavan et al., 2006; Chang et al., 2007; Colombo et al., 2008; Mawase et al., 2011; Bourke et al., 2015; Gilliaux et al., 2015).

**Table 2:** **Parameters and underlying measures used in the reviewed studies for assessment of motor learning, and implemented clinical tests**.

**Study**	**Motor learning parameters**								**Clinical measures and the timing of their assessment**
	**Adaptation (*N =* 6)**	**After-effects (*N =* 3)**	**De-adaptation (*N =* 4)**	**Anticipatory control (*N =* 2)**	**Performance (*N =* 24)**	**Acquisition (*N =* 8)**	**Retention (*N =* 8)**	**Transfer (*N =* 14)**	
Aluru et al., 2014	–	–	–	–	Movement speed; Electromyographic activity: movement amplitude and muscles activation	–	–	–	FMA; MAS; Joint proprioception threshold; GDS; BRUMS (Pre-intervention)
Boyd and Winstein, 2001	–	–	–	–	Median reaction time	–	–	–	MMSE (Pre-intervention)
Boyd and Winstein, 2004	–	–	–	–	RMSE: errors; Spatial-temporal accuracy	–	RMSE: errors; Spatial-temporal accuracy	–	MMSE; FMA (Pre-intervention)
Boyd and Winstein, 2006	–	–	–	–	Median response time; RMSE	–	Median response time; RMSE	–	MMSE; FMA (Pre-intervention)
Bourke et al., 2015	Posture speed; Deceleration time; Maximal displacement; Return time to baseline position; Endpoint error; Multi-joint coordination	–	–	–	–	–	–	–	CMSA; Muscle power of elbow and shoulder; FIM (Pre-intervention)
Burtner et al., 2014	–	–	–	–	RMSE; RMSE variability	RMSE; RMSE variability	RMSE; RMSE variability	–	MVPT; BBT (Pre-intervention)
Caimmi et al., 2008	–	–	–	–	Movement duration; End of movement angle; Mean angular velocity; MS; Mean target approaching velocity	–	–	–	WMFT; MAL; QIOM; AOU of affected hand; BI; ESS (Pre and post intervention)
Casadio and Sanguineti, 2012	–	–	–	–	Speed; Precision; MS; Dependence of voluntary control on previous and next trials; Assistance rate; Noise Vision bias	–	Correlation between the dependence of voluntary control on previous trials and the percentage change in FMA after 3 months	–	MAS; FMA (Pre and post intervention, correlations with motor learning measures)
Chang et al., 2007	–	–	–	–	PV; %TPV; MT; Normalized jerk score	–	–	–	FMA; MAS; FAT; Strength: isometric grip and push and pull (Pre and post intervention)
Chen et al., 2012	–	–	–	–	RT; NMT; NMU; PV	–	–	–	PMAL; MAS; PMDS (Pre and post intervention, correlations with motor learning measures)
Chen et al., 2014	–	–	–	–	RT; NMT; MS; PV; MGA; Percentage of movement where MGA occurs	–	–	RT; NMT; MS; PV; MGA; Percentage of movement where MGA occurs	BOTMP; Fine motor domain of PDMS-2; WeeFIM (Pre and post intervention, correlations with motor learning measures)
Christopher and Johnson, 2014	–	–	–	–	Time to completion; MS	–	–	–	FMA (Pre-intervention)
Colombo et al., 2008	–	–	–	–	–	AMI; MD from theoretical path; nPL; MS; MT; Force control	–	–	MPS; MSS; FMA (Pre and post intervention, correlations with motor learning measures)
Cirstea and Levin, 2007	–	–	–	–	Angular motions; TCI; Trunk's displacement	–	Angular motions; TCI; Trunk's displacement	Same measures as for performance and retention in a different workspace	CMSA; FMA; CSI; TEMPRA (Pre and post intervention, correlations with motor learning measures)
Dancause et al., 2002	Angular positions; Angular torques	–	–	–	–	–	–	–	CMSA; CSI; FMA; Neuropsychological testing (Pre-intervention)
Dipietro et al., 2007	–	–	–	–	–	–	–	Axes ratio; Joint angles correlation metric; Orientation; Major and minor axes of ellipse	MMSE; MPS; FMA (Pre and post intervention)
Dipietro et al., 2009	–	–	–	–	–	–	–	Axes ratio; MS; Submovements	MMSE; MPS; FMA (Pre and post intervention)
Dipietro et al., 2012	–	–	–	–	Mean speed; PV; MS; Movement duration; Submovements	–	–	Axes ratio	MMSE; FMA (Pre and post intervention)
Durham et al., 2014	–	–	–	–	Movement duration; MS PV; %TPV; %TPD; %TPA; Peak aperture size; Peak elbow extension	–	–	–	FMA; Birmingham University Cognitive scale (Pre-intervention)
Geerdink et al., 2013	–	–	–	–	–	BBT	–	–	MACS; AHA; Melbourne Assessment COPM (Pre and post intervention)
Gilliaux et al., 2015	–	–	–	–	Straightness amplitude; CV of straightness; Speed index; CV of jerk; CV of speed	–	–	–	BBT; MACS; QUEST; MAS; Elbow flexors and extensors strength; Muscle torques; Abilhand-Kids (French version); PEDI; Life Habits questionnaire (French version) (Pre and post intervention)
Hemayattalab and Rostami, 2010	–	–	–	–	–	Proximity to a target	–	Proximity to a target	
Hemayattalab et al., 2013	–	–	–	–	–	Proximity to a target	–	Proximity to a target	GMFCS (Pre-intervention)
Kitago et al., 2013	–	–	–	–	–	–	MT; PV; Initial directional error; Path curvature; Systematic error; Submovements	–	ARAT; FMA (Pre and post intervention)
Kitago et al., 2015	–	–	–	–	–	–	Same measurement as for transfer, three weeks post training	MT; Reaching trajectories; Directional error; End-point accuracy; MS; Trajectory analysis	FMA; ARAT (Pre and post intervention, correlations with motor learning measures)
Krebs et al., 2012	–	–	–	–	Deviation from a straight line; MT; PV; Movement duration; MS	–	–	Axes ratio	FMA; QUEST; MAS; Parent questionnaire (Pre and post intervention)
Masia et al., 2011	Lateral deviation from straight line; Acceleration; peak speed; Peak average speed; Anisotropy index	Same as for adaptation	Same as for adaptation	–	Change of lateral deviations throughout training	–	–	–	MAS; FMA; Melbourne scale (Pre-intervention)
Massie et al., 2009	–	–	–	–	–	Trajectory variability; MT; Reach velocity; Shoulder abduction angle; Segmental contribution of trunk, shoulder and elbow	–	–	MMSE; MAL; McGill Pain Scale; WMFT (Pre and post intervention)
Mawase et al., 2011	–	–	–	Grip force; Vertical trajectory; Temporal coordination	–	–	–	–	MACS; JTT (Pre and post intervention, correlations with motor learning measures)
Molier et al., 2011	–	–	–	–	–	Average deviation from predefined path; Change in difficulty level	–	Elbow and shoulder joints: excursions, positions and coordination	FMA; MI; ARAT
Orrell et al., 2007	–	–	–	–	Median response time	–	Median response time	Median response time	MMSE; Functional Impairment Questionnaire (Pre-intervention)
Patton et al., 2006	Average shift in initial direction	Initial direction error	Initial direction error	–	Reduction in initial direction error	–	–	–	
Pohl et al., 2006	–	–	–	–	Mean response time; CV of response time	–	–	–	MMSE; Lighthouse Near Acuity Test; Ishihara's Test; Florida Apraxia Screen (Pre-intervention)
Raghavan et al., 2006	–	–	–	Peak load force; Timing and efficacy of grip load force coordination	–	–	–	–	FMA; MMSE; Two-Point Discrimination Test; MAS; WMFT; PPT (Pre and post intervention, correlations with motor learning measures)
Schaefer et al., 2013	–	–	–	–	Number of successful trials	–	–	BBT; Number of buttons fastened/ unfastened; Flexion angle of shoulder and elbow; Hand path; Dual task	Maximum grip strength; ARAT; MAS; The Trail-Making Test (Pre-intervention)
Scheidt and Stoeckmann, 2007	Initial direction error; End point accuracy; Movement onset speed; Peak speed point; Penultimate position point; Final position point	–	Persistence of the compensatory response acquired during training	–	–	–	–	–	FMA; MAS; ROMs (shoulder, elbow and wrist) Proprioception (thumb, wrist, elbow, and shoulder) (Pre-intervention)
Senesac et al., 2010	–	–	–	–	–	–	–	Hand path curvature; Time to peak velocity; PV; MS; Acceleration	MAS; WMFM; FMA; MAL (Pre and post intervention, correlations with motor learning measures)
Sterpi et al., 2012	–	–	–	–	–	–	–	AMI; MT; MD; nPL; MS	
Takahashi and Reinkensmeyer, 2003	Spatial reaching error; Late correction	Same as for adaptation	Same as for adaptation	-	Difference in error between 1^*st*^ and 5 last reaches	–	–	–	CMSA (Pre-intervention)
Thaut et al., 2002	–	–	–	–	–	Movement duration; Variability of timing; Reaching trajectories; Rhythmic synchronization	–	–	Brunnstrom-Scale; ROM (elbow, shoulder) Dexterity (Pre-intervention)
Wu et al., 2007	–	–	–	–	RT; PV; MT Total displacement	–	–	–	FMA; MAL (Pre and post intervention)
Wu et al., 2011	–	–	–	–	NMT; NMU; PV; MS; Percentage of movement time where peak velocity occurred	–	–	–	WMFT; MAL (Pre and post intervention)

*“pre and post intervention” in the “Clinical measures and the timing of their assessment” column relates to at least one, but not necessarily all the clinical measures, as some were only assessed for establishing the inclusion criteria*.

*Abbreviations: FMA, Fugl-Meyer Assessment; MAS, Modified Ashworth Scale; GDS, Geriatric Depression Scale; BRUMS, Brunel Mood Scale; MMSE, Mini Mental State Examination; RMSE, Root Mean Square; CMSA, Chedoke-McMaster Stroke Assessment Scale; FIM, Functional Independence Measure; MVPT, Motor-Free Visual Perception Test; BBT, Box and Blocks Test; WMFT, Wolf Motor Function Test; MAL, Motor Activity Log; QIOM, Quality Of Movement; AOU, Amount Of Use; BI, Barthel Index; ESS, European Stroke Scale; MS, Movement Smoothness; PV, Peak Velocity; %TPV, Percentage Time to Peak Velocity; MT, Movement Time; FAT, Frenchay Arm Test; RT, Reaction Time, NMT, Normalized Movement Time; NMU, Normalized number of Movement Units; PV, Peak Velocity; PMAL, Pediatric Motor Activity Log; PDMS, Peabody Developmental Motor Scale; MGA, Maximum Grip Aperture; BOTMP, Bruininks-Oseretsky Test of Motor Proficiency; WeeFIM, Functional Independence Measure for children; MD, Mean Distance; AMI, Active Movement Index; nPL, Normalized Path Length; MPS, Motor Power scale; MSS, Motor Status Score; TCI, Temporal Coordination Index; CSI, Composite Spasticity Index; TEMPRA, Upper Extremity Performance Test for the Elderly; %TPD, Percentage to Peak Deceleration; %TPA, Percentage to Peak Aperture; MACS, Manual Ability Classification System; AHA, Assisting Hand Assessment; Melbourne Assessment, Melbourne assessment of Unilateral Upper Limb Function; COPM, Canadian Occupational Performance Measure; QUEST, Quality of Upper Extremity Skills Test; PEDI, Pediatric Evaluation of Disability Inventory; GMFCS, Gross Motor Function Classification System; ARAT, Action Research Arm Test; JTT, Jebsen-Taylor test; MI, Motricity Index; PPT, Purdue pegboard test; ROM, Range Of Motion*.

The synthesized data provides an outline of the various parameters utilized in the studies for assessment of motor learning through an overview of each study. Parameters are emphasized in **bold** throughout the text. Measures used to assess the parameter are stated for each study, and underlined throughout the text.

### Assessment of adaptation and anticipatory control

Six of the reviewed studies examined the **adaptation** process (Dancause et al., 2002; Takahashi and Reinkensmeyer, 2003; Patton et al., 2006; Scheidt and Stoeckmann, 2007; Masia et al., 2011; Bourke et al., 2015). Two studies assessed the predictive control (Raghavan et al., 2006; Mawase et al., 2011).

Patton et al. (2006) and Takahashi and Reinkensmeyer (2003) evaluated stroke subjects performing robotic reaching movement training. A baseline phase without perturbations was followed by a learning phase with constant exposure to forces. Then, **after-effects** were measured by a catch trial phase that included intermittent removal of the force field. Finally, when forces were completely removed (i.e., washout period), **de-adaptation** was assessed (i.e., tendency of the after-effects to disappear). Patton et al. (2006) measured the initial direction error, testing the effectiveness of the shift of the early part of the movement, and the average shift in initial direction from the un-perturbed baseline trials to the after effects catch trials to establish the **adaptation capacity**. Takahashi and Reinkensmeyer (2003) also assessed the **adaptation capacity** of stroke subjects by the spatial reaching error from baseline. But the late correction (the distance from the maximal and final deviation from the reference path) was also measured, to assess the whole movement following applied and removed force fields. **Performance improvement** was assessed as the reduction in reaching error from baseline to final phase (Takahashi and Reinkensmeyer, 2003; Patton et al., 2006). Scheidt and Stoeckmann (2007) examined the **adaptation** to velocity dependent perturbations with pseudo-random magnitude, using measurement of the initial direction error, and the end point accuracy. The **compensatory response** acquired during training was measured by the hand movement onset speed, peak speed point, penultimate position point (i.e., the moment the speed dropped below 20% of its maximal value) and by the final position point. Dancause et al. (2002) explored **error correction strategies** following an unexpected spring-like load presented in 30% of elbow flexion movement trials. The strategies were identified by comparison between the angular positions and torques of the initial movement before the load was applied and the correction following the load. Bourke et al. (2015) assessed the **corrective responses** after unexpected external perturbations to the elbow or shoulder, while instructed to maintain their hand at a spatial goal. The response was quantified by the posture speed before the perturbation, deceleration time and maximal displacement after perturbation, return time to baseline position, end point error and by the joint velocity offset that represents the multi-joint coordination. Masia et al. (2011) explored the ability of children with CP to **adapt** to randomized center-out movements while performing reaching movements toward peripheral targets. For each reaching movement lateral deviation from straight line, acceleration peak, and peak and average speeds were measured at familiarization, force field adaptation, and washout phases. Directional analysis was performed for all measures to assess the anisotropy index (i.e., the roundness or flatness of the ellipse). The **learning index** was established as **the degree of adaptation** measured by the lateral deviations in force field and catch trials.

Mawase et al. (2011) explored the **predictive control** of CP subjects using a grasp and lift task of a virtual object. A sequence of increasing weights appeared randomly within trials of random weights. **The planning of grasp precision** was assessed by measurement of the grip force at the beginning of the lifting task and by the vertical trajectory of the object estimating the motor command. **The precision of grasp execution** was assessed by measurement of the temporal coordination. Raghavan et al. (2006) also explored the **planning of precision grasp** and **precision of grasp execution** among stroke patients. The peak grip and load forces and the timing and efficacy of grip load force coordination were measured respectively.

### Assessment of performance and skill acquisition

Chen et al. (2012) examined the reaching **performance** of children with CP by measurement of reaction time (RT), normalized movement time (NMT), normalized number of movement units (NMU) and the peak velocity (PV). Three studies assessed the **performance** of stroke patients performing a reaching movement task following an intervention (Wu et al., 2007; Caimmi et al., 2008; Durham et al., 2014). Wu et al. (2007) measured the RT, PV, movement time (MT) and the total displacement. Caimmi et al. (2008) measured the movement duration, end of movement angle, mean angular, and target approaching velocities, consistency of the target approaching and the movement smoothness (MS). The study objectives were to examine whether kinematic analysis is a sensitive and reliable measure, whether it can identify the mechanisms leading to improvement and quantify the functional improvement. Durham et al. (2014) measured the movement duration, PV, percentage time to peak velocity (%TPV), or to peak deceleration and to peak aperture, peak aperture size, peak elbow extension, and the MS. Wu et al. (2011) assessed the **performance** of stroke patients during pressing a desk bell and pulling a drawer tasks by measurement of the NMT, NMU, PV, percentage of movement time where peak velocity occurred, and the MS. Christopher and Johnson (2014) examined the **performance** of stroke patients performing a drinking task by measuring time to completion and the MS. Aluru et al. (2014) evaluated the **performance** of stroke subjects by measuring the movement speed, electromyographic activity of wrist extension, activation of wrist extensor and flexor, and co-activation of antagonist muscles during a wrist flexion-extension task.

While Wu et al. (2007), Aluru et al. (2014), Caimmi et al. (2008), Christopher and Johnson (2014), and Durham et al. (2014) studied only the performance, which is an individual execution of a skill (Krakauer and Mazzoni, 2011; Kitago and Krakauer, 2013), four studies (Thaut et al., 2002; Colombo et al., 2008; Massie et al., 2009; Geerdink et al., 2013) explored the **skill acquisition**, which depends on extended practice of the performance (Krakauer and Mazzoni, 2011; Kitago and Krakauer, 2013). Colombo et al. (2008), Thaut et al. (2002) assessed the **performance change** of stroke patients' reaching movements. Colombo et al. (2008), with the purpose of better understanding the learning mechanisms of stroke patients, measured the efficacy [the active movement index (AMI)], accuracy [mean distance (MD) from theoretical path], efficiency [normalized path length (nPL)], MS, MT, and the force control (error in orientation of force generation) of reaching movements. Massie et al. (2009) measured the trajectory variability, MT, reach velocity of reaching movements and the difference in compensatory strategies during treatment. Thaut et al. (2002) measured the movement durations, arm kinematics, variability of timing, reaching trajectories, and rhythmic synchronization. Geerdink et al. (2013) assessed the **learning curve** of manual dexterity of children with CP to establish the time during the intervention when maximal effects were reached using the box and block test (BBT) (counts the number of blocks that are transferred with a single hand from one compartment to another within 60 s).

### Assessment of transfer and retention

Retention and transfer tests vary in the information received regarding the obtained learning. The transfer test assess a skill that was not practiced, whereas the retention test examines the trained task after a time interval (Schmidt and Lee, 2004; Kantak and Winstein, 2012).

Six studies explored whether trained movement **generalized** to untrained movement using **transfer** tests (Dipietro et al., 2007, 2009; Senesac et al., 2010; Dipietro et al., 2012; Krebs et al., 2012; Kitago et al., 2015). Dipietro et al. (2007, 2009, 2012), and Krebs et al. (2012) examined the **generalization** of trained reaching movements to untrained circle drawing movements by measurement of the axes ratio metric (i.e., indication for shoulder-elbow coordination). Additionally the studies measured speed profiles, MS, and measures of submovements [i.e., discrete ballistic movements that are a part of a more complex movement (Rohrer et al., 2004)] (not all measures were used in all studies, see Table 2). Dipietro et al. (2007) also examined changes in flexor-extensor abnormal synergies by measuring the joint angles correlation metric (independence of elbow and shoulder movements), orientation (best fitting line of hand path) and the major and minor axes of the drawn ellipse. Senesac et al. (2010) assessed the spatial **generalization** of improved proximal inter-joint coordination to two untrained reaching tasks, one spatially similar and the other different. Reach end-point kinematics (hand path curvature, time to peak velocity, PV, MS, and acceleration) were measured. Kitago et al. (2015) measured the reaching trajectories and their quality (i.e., trajectory analysis), MT, directional error, MS and and-point accuracy to assess the **transfer** of trained goal-directed reaching movements to untrained out-and-back straight movements. Two studies suggested that motor recovery after stroke and motor habilitation of children with CP resembles a motor learning model more than an adaptation model, as the trained movements **generalized** to the untrained movements (Dipietro et al., 2012; Krebs et al., 2012).

While previously discussed studies implemented transfer tests assessing the generalization from trained to untrained movements (Dipietro et al., 2007, 2009, 2012; Senesac et al., 2010; Krebs et al., 2012; Kitago et al., 2015), Sterpi et al. (2012) implemented a transfer test that assessed the **generalization** of movements trained in a certain workspace (reaching movements to form a path of a square) to a different workspace (within and outside the square) of stroke subjects. The AMI, MT, MD (error of movement accuracy), nPL (error of movement efficiency) and the MS were measured. Gilliaux et al. (2015) examined the **performance** of children with CP by measuring the amplitude and coefficient of variation (CV) of straightness for a reaching-as-far-as-possible task, the speed index metric for reaching toward a target task, and the CV of jerk and speed metrics for drawing a square and a circle tasks. In addition, BBT and a wide range of clinical and functional measures of activity and participation were performed (see Table 2). As some of the tasks included movements that were not trained, the described measures were also included as part of the **transfer** parameter (Table 2). Schaefer et al. (2013) measured the **performance** during a feeding task by measuring the number of successful repetitions, defined as spooning and transferring at least one bean from one cup to another. **Transfer** was assessed by sorting (BBT, spatiotemporally similar), dressing (spatiotemporally different), and dual tasks. The dual task conditions assessed whether automaticity transferred across tasks, measured by the difference between the reported and correct number of times a letter was heard in a sequence of letters.

Four studies examined the effect of different feedback frequencies on motor skill learning (Cirstea and Levin, 2007; Hemayattalab and Rostami, 2010; Hemayattalab et al., 2013; Burtner et al., 2014). Burtner et al. (2014) assessed the **performance accuracy** and **consistency** of an elbow extension-flexion reversal movement, which were assessed separately on **acquisition** at first day, **retention** (without feedback) and **reacquisition** (with feedback) on the second day. **Accuracy** was measured using the root mean square error (RMSE) (i.e., the average difference between the goal movement trajectory and the participants response), and **consistency**, by the variability of the RMSE. Hemayattalab and Rostami (2010) and Hemayattalab et al. (2013) evaluated new motor skill learning of children with CP—darts and bean-bags throwing tasks. The **accuracy** of scores was measured by the proximity to a center-peripheral target at **acquisition** and at **retention**, 3 days after practice. Cirstea and Levin (2007) examined the **performance** and **retention** following 1 month of pointing movements training to the contralateral workspace by measurement of the angular motions of the elbow and shoulder joints, the elbow-shoulder interjoint coordination and by the amount of trunk's anterior and rotational displacement. A **transfer test** included pointing movements toward an ipsilateral target.

Molier et al. (2011) examined the effect of position resistance feedback, provided when a deviation from a predefined path occurred, during three reaching task trainings (moving hand; making a curve; lifting hand to shelf). The average use of the feedback was calculated, and the difficultly level was established by measurement of the reached height and diameter of predefined path. In addition, elbow and shoulder joint excursions, positions, and coordination, and an isometric strength task for which the maximal voluntary torque (MVT) were measured during a task of circular arm movements. The parameters that were utilized to assess learning were not directly specified in the study. Therefore, as the circular movements were not trained, we placed the measures under the **transfer** principle (see Table 2). The measured feedback frequency and difficulty level during training corresponds with the **performance change** that also may be addressed as the **acquisition** parameter.

Kitago et al. (2013) aimed to determine the feasibility in implementing kinematic measurements (MT, PV, absolute initial directional error, path curvature, systematic error, number of submovements) of arm reaching and wrist pointing tasks, and of clinical measures for understanding the motor recovery process within 2 weeks after CIMT (i.e., **retention**. Chang et al. (2007) assessed the **performance** and **retention** of stroke subjects after reaching training, by measuring the PV, %TPV, MT, normalized jerk score, and limb muscle strength. Chen et al. (2014) assessed the **performance** and **retention** at 3- and 6-months by examining the speed and dexterity during 8 object manipulation tasks (Bruininks, 1978), by functional ability measures (see Table 2) and by kinematic analysis (RT**, NMT, MS, PV, maximum grip aperture (MGA), and the percentage of movement where MGA occurs) during a reach-to-grasp task. Casadio and Sanguineti (2012) examined the **performance change** of stroke patients over a robot-assisted arm extension task practice. Performance measures (i.e., speed, precision, and smoothness), retention rate (dependence of voluntary control on previous trials), learning rate (dependence of voluntary control of next trials on current trial), assistance rate, noise (voluntary control not accounted for learning) and vision bias were measured. **Retention** was examined by estimating the correlation between the retention rate during performance to the percentage change in the FMA 3 months post the rehabilitation trial.

### Motor sequence learning

Five studies assessed motor sequence learning of stroke patients (Boyd and Winstein, 2001, 2004, 2006; Pohl et al., 2006; Orrell et al., 2007). Two of them included practice of the serial reaction time task (SRTT), which includes a movement to press one of four targets when cued. When the correct key is pressed, the next cue was delivered (Boyd and Winstein, 2001; Orrell et al., 2007). Boyd and Winstein (2001) assessed learning by **performance change** over practice of the median reaction time, whereas Orrell et al. (2007) assessed the median response time both during **performance** and **retention**. Orrell et al. (2007) also implemented two **transfer** tests, once by changing the motor sequence and by changing the required movement from index finger only to whole arm movement. Pohl et al. (2006) examined the **performance change** during practice of an implicit motor learning task of stroke patients undergoing a motor task with random and repeated sequences by the mean response time and by the CV of response time. To examine the **performance** following practice, subjects were requested to perform the sequence practiced in the repeated conditions once again. In one study (Boyd and Winstein, 2004) subjects practiced the continuous tracking (CT) task that included tracking of the vertical path of a target cursor. The middle third of each tracking trial was repeated, whereas the first and last third were random. Reduction in tracking errors measured by the RMSE and spatial-temporal accuracy were measured to assess **performance change** over practice and **retention** (Boyd and Winstein, 2004). In the final study subjects practiced both the SRTT and CT task (Boyd and Winstein, 2006). Learning was inferred by the median response time and RMSE respectively, at **performance** and **retention**.

## Discussion

The purpose of this review was to identify the different parameters and the variety of measures utilized in the literature to assess motor learning, and to categorize them based on the learning type and process they examine, while taking into consideration the patients' features and the studies' methodology and intervention. Over the past 15 years, 42 studies were identified as directly assessing the learning process of persons following stroke or with CP following different interventions. The studies varied by the parameters and measures utilized to infer motor learning. A sensible selection of an outcome measure is an important part when planning an intervention. The questions we raised were the studies' methods for selection of the relevant parameters and metrics, the differences between the measures and the information each metric can provide.

### Measures of adaptation vs. skill learning

Adaptation was assessed in the reviewed studies using dynamic perturbations by induced force fields during reaching movements (Dancause et al., 2002; Takahashi and Reinkensmeyer, 2003; Patton et al., 2006; Scheidt and Stoeckmann, 2007; Masia et al., 2011). Despite the variability in methodological designs (e.g., practice—number of trials and repetitions, utilized measures, etc.) between the studies, all induced a change in environment and assessed the resulted change in behavior. The manifestation of the adaptation parameter, in all studies, was evaluated by some sort of measurement of the extent and/or quickness in which the performance returned to the pre-perturbation level and of the sensory-prediction errors reduction. The changes in the after-effects assess the update of the internal model (Krakauer, 2006; Huang and Krakauer, 2009). Robots were described as suitable to record movement data (e.g., position, velocity, and joint torques), which allows quantitative reliable measurement of kinematics and dynamics during recovery (Huang and Krakauer, 2009).

The utilized measures and methods of their implementation can affect the obtained results. For example, Patton et al. (2006) concluded that stroke survivors preserved the ability to adapt, whereas Takahashi and Reinkensmeyer (2003) found their adaptive capability to be reduced. Their different results may be explained by the fact that Patton et al. (2006) utilized a metric that measured only the early part of the movement, whereas Takahashi and Reinkensmeyer (2003) assessed the whole movement. Evaluation of the anticipatory control should include a time limit for the movements performed. The limit's purpose is to minimize online corrections and focus on deficits in the feed-forward mechanism (Kitago et al., 2013). For example, Raghavan et al. (2006) estimated the predictive control of children with CP by estimation of the motor command at the first 70 ms of a grasp and lift task. Without the time limit, the prolonged time they needed to receive sensory feedback for grip force generation, would not have been identified (Raghavan et al., 2006). Contrarily, the findings of Patton et al. (2006) cannot serve as a measure of feed-forward control error as it does not consider the time between the feed-forward control and movement initiation (Patton et al., 2006).

It is interesting to note that only studies that explored the anticipatory control utilized measures of force generation, while other studies used only kinematic measures. This is true of all but four of the reviewed studies that did include force components as part of their measurements (Chang et al., 2007; Colombo et al., 2010; Bourke et al., 2015; Gilliaux et al., 2015). Takahashi and Reinkensmeyer (2003), who did not measure movement dynamics, suspected that the rate of force development explains the impaired anticipatory control of the paretic arms. Measurement of the force generation may clarify whether the deficit in the anticipatory control is due to inability to form internal models or to implement them (Takahashi and Reinkensmeyer, 2003).

Scheidt and Stoeckmann (2007) found that stroke patients adapt similarly to healthy individuals; however, they may require more practice, as they had more influence of prior error on subsequent movement. Dancause et al. (2002) added that severely affected individuals might require more practice, as they required more trials to diminish errors than mildly affected individuals. It can be inferred that measurement of the rate at which the errors decrease throughout trials and the influence of the prior error on the next movement, can be used as measures to evaluate the amount of practice an individual will require.

Adaptation may be an initial ingredient in a motor learning model (Bastian, 2008), and was implied to be a form of implicit learning that requires no awareness (Krebs et al., 2001). However, skill acquisition often requires conscious awareness and practice of the performance (Krakauer and Mazzoni, 2011; Kantak and Winstein, 2012). Performance curves indicating change throughout practice can be established for a variety of measures. Geerdink et al. (2013) used the performance curve of manual dexterity improvement to evaluate the point in time where maximal effects are learned and achieved, finding that age has an effect on the speed of dexterity gain. It can be inferred from these results that the learning curve can be used as a measure to study and evaluate the maximal effect of various interventions, which might later on assist to better establish individualized training timings to fulfill maximal potential.

None of the studies that examined the adaptation parameter assessed the persistence of the after effects. Measures of performance can be implemented at different times throughout the training, but can also be used at a time interval after the last practice to assess the retention. As performance might be affected by transient factors, such as feedback, attention, fatigue etc., performance measures may be limited to the acquisition phase. Therefore, a retention test is preferable to infer learning, as it assess long-lasting changes indicating constancy of the level of performance achieved at acquisition and strength of the motor memory (Schmidt and Lee, 2004; Kantak and Winstein, 2012).

Another important aspect of learning is the extent to which what was learned during practice generalizes outside of practice settings (Schmidt and Lee, 2004; Kantak and Winstein, 2012), assessed with transfer tests and reflects the flexibility of the motor memory (Schmidt and Lee, 2004; Kantak and Winstein, 2012). According to the motor learning theory of Fitts and Posner (1967), presenting a three stage model of learning—cognitive, associative, and autonomous, achieving transfer (automatization) and retention of a skill is necessary to indicate learning has occurred (Cano-de-la-Cuerda et al., 2015). It was suggested that when the trained and untrained tasks share similar neural demands, generalization to the untrained task is increased (Sainburg and Wang, 2002; Schmidt and Lee, 2004; Shadmehr, 2004). This implies that the transfer test should encompass and examine similar motor control requirements as the training. The transfer parameter is essential, since the rehabilitation aim is to grant the patient optimal function and independence, only possible when improvements generalize to real-life situations.

Some prominent theories of motor learning require a comprehensive analysis of movement that includes many of the measurements used in the reviewed studies (Cano-de-la-Cuerda et al., 2015). However, most of these studies did not qualify to infer learning according to these theories. For example, parameters and measures implemented during tasks with restriction on degrees of freedom contradict Bernstein's model of motor learning (Bernstein, 1967) that requires the learner to increase the degrees of freedom as an indication of learning. Similarly, many of the studies evaluated the efficiency and consistency of the movement without testing transfer and *vice versa*. Both are essential to demonstrate learning according to Gentile's theory of motor learning (Gentile, 1972). Therefore, studies who aim explicitly to demonstrate learning must confide to a theory, and take it into consideration when designing the study.

### Measures of recovery vs. compensation

Improved activity can arise from either recovery of impairment, development of compensatory movements, or both. There is a necessity to differentiate between impairment of execution and impaired motor learning (Boyd and Winstein, 2004). The implemented settings and outcome measures of a study may affect the interpretation of the results. Patton et al. (2006) supported the hand against gravity, which could decrease the effect of the impairment in movement execution (due to weakness, spasticity etc.) and result in detection of adaptive capability. On the contrary, Takahashi and Reinkensmeyer (2003) did not support the hand, which might led to poor adaptive capability among subjects, when in fact execution impairment increased due to gravity.

The state of mind presented in a study by Nourrit-Lucas et al. (2013), who explored long term retention of neurologically intact subjects, might also be relevant to studies performed on neurologically impaired individuals. The authors suggested that the experimental settings in which the learning is assessed, affects the measurements and the conclusions drawn regarding the learning process. Performance variables usually assess simple tasks with few degrees of freedom. They are defined as parameters measuring performance in terms of speed and accuracy, representing the outcome of the behavior with respect to the goal of the task (Nourrit-Lucas et al., 2013). A more complex task has a larger number of degrees of freedom, and learning can be explored by coordination variables measuring the spatiotemporal functional organization between body segments in terms of phase relations (Kelso, 1997; Nourrit-Lucas et al., 2013). In most of the reviewed studies, simple tasks were examined, among the rest, to decrease the probability for compensation. Moreover, in approximately half of the discussed studies the arm was supported against gravity, reducing even more the degrees of freedom. Retention tests assess the same coordinative pattern: an improvement is expected due to the motor plan that was established during practice, whereas a transfer test would examine the adaptability of the motor program. Therefore, transfer tests are more suitable to examine the coordinative measures (Nourrit-Lucas et al., 2013). The coordinative improvement can represent the reacquisition of the pre-lesion patterns, addressed as recovery (Levin et al., 2009). None of the reviewed studies assessed the coordination of a more complex task.

Kinematic analysis of various sorts for measurement of parameters were performed by all but three of the reviewed studies (Hemayattalab and Rostami, 2010; Geerdink et al., 2013; Hemayattalab et al., 2013). Subramanian et al. (2010) separated the kinematic variables to measures of motor performance and to measures of movement quality. Two of the discussed performance measures that were often utilized in the reviewed studies to assess the parameters of motor skill learning are measures of accuracy and velocity of movement. Reduction in errors of these measures indicates improvement of performance (Krakauer, 2006; Schmidt and Wrisberg, 2008). However, in motor execution of a task there is a relationship between movement speed and accuracy, and depending on task requirements, the accuracy or speed component can be prioritized (Fitts, 1954; Kitago and Krakauer, 2013). If only one of them is assessed, an improvement does not necessarily indicate an improved skill. For example, a subject can make more errors as speed increases, or slow down for a more accurate movement (Kitago and Krakauer, 2013). The movement quality measures include the configuration of the examined limb and measurement of compensatory movements, and were suggested as able to distinguish between recovery and compensation (Subramanian et al., 2010). Almost half of the reviewed studies restricted compensatory movements, preventing examination of movement quality required in real-life settings. How then, can complex motor skills be assessed in terms of qualitative organization for neurologic patients with a variety of heterogenic impairments, potentially masking learning? In some of the reviewed studies the learning following a treatment was inferred by examination of the less affected limb (Table 1). This may distinguish motor execution impairments due to hemiparesis, which may mask motor learning, from deficient motor learning (Boyd and Winstein, 2004).

Many studies evaluate the performance ability of their subjects using functional tests (Kitago and Krakauer, 2013). Functional tests often include more complicated movements with multiple degrees of freedom, resemble real-life activities, and are categorized under the “activities” domain of the ICF classification (Sivan et al., 2011). However, clinical measures do not consider the quality of the movement, and therefore do not measure a decrease in impairment or return to a normal motor control (Kitago et al., 2013). Therefore, functional improvement in clinical measures, but not in measures of impairment (such as the FMA and kinematic analysis), can be attributed to compensatory strategies (Kitago et al., 2013). Kinematic variables were suggested to be valid for differentiating compensation from recovery and for measurement of upper limb impairment (Subramanian et al., 2010). Huang and Krakauer (2009) reviewed rehabilitation strategies and outcome measures for impairment versus function. They suggested that in the acute and sub-acute stages of recovery, rehabilitation treatment should focus on the impairment level while refraining from compensatory adjustments, and only after a certain level of improvement is achieved, focus on functional performance. It can be inferred that the measure of assessment should also be fitted to the stage and timing within the recovery process, and should be able to distinguish between them. Movement quality measures can be sensitive and useful for complementing clinical assessment (Subramanian et al., 2010). Moreover, Sivan et al. (2011) suggested that when evaluating a rehabilitation program it is important to measure each domain within the ICF classification. While most clinical tests were classified as evaluating the “activities” domain, most of the measures that we reviewed, such as kinematic analysis and the BBT, were placed under assessment of the body functions domain. This different classification might explain the absence or low correlation between the measures in some of the studies, as they might assess different features of learning. This implies that when assessing motor learning measures different features should be taken into consideration.

### Reliability and validity of outcome measures

The validity and reliability are important components for a meaningful behavioral research. The validity represents the degree to which a study measures what it intends to measure, and reliability is the consistency of the results (Forzano and Gravetter, 2009). Therefore, both the validity and reliability are important in order to infer whether the parameters and measures used in the study provide accurate representation of the change during and after practice.

Kinematic movement quality measures were found to be valid and sensitive for recognizing upper limb impairments of stroke patients performing pointing and reach-to-grasp tasks (Subramanian et al., 2010). However, as mentioned previously, some of the reviewed studies did not examine quality measures, due to restriction of the upper limb during movement and of compensatory movements, or due to measurement of solely motor performance measures.

While the validity and reliability of the clinical measures were mostly stated in the reviewed articles, these were less addressed for the motor learning parameters and laboratory-based measures. Some studies mentioned the objective behind their motor control outcome measures. For example, Gilliaux et al. (2015) and Durham et al. (2014) measured kinematics previously established and described sensitive, respectively Geerdink et al. (2013) used the BBT because of its feasibility, validity and reliability (Jongbloed-Pereboom et al., 2013). Other studies focused on examining the strength of their measures. Bourke et al. (2015) found good to excellent reliability coefficients for task performance measures. Kinematic analysis was described as sensitive for motor control measurement (Chen et al., 2012), and for evaluation of motor recovery (Caimmi et al., 2008). Kitago et al. (2015) suggested that their method of analyzing reaching kinematics, based on functional principal component analysis (Yao et al., 2005; Goldsmith et al., 2013), is more sensitive than measures such as the end-point accuracy or PV kinematic measures due to its additional ability to evaluate the entire trajectory of movement.

Across the reviewed studies, not all parameters were equally studied and the reasoning for parameter selection was not addressed in all but one study. The validity and reliability of the assessed parameters were also not addressed. Kantak and Winstein (2012) suggested that retention and transfer tests, rather than solely the performance parameter, should be implemented to negate transient performance changes and detect motor learning. In this review, the performance was most often assessed, in 24 of the reviewed studies, whereas transfer was evaluated in 14, and retention in only eight of the studies. Retention was examined following a time interval in all studies, but the length of the retention interval varied. Kantak and Winstein (2012) categorized retention into immediate and delayed (i.e., implemented at least 24 h after practice), and suggested that different retention intervals can yield different conclusions regarding the obtained learning (Kantak and Winstein, 2012).

### Review's limitations

This review has several limitations. Firstly, we focused only on studies of the upper limb. As the lower limb role and function differs from the upper limb, it is important to further address the metrics utilized for assessment of the lower extremity with relation to their place within the motor learning model. Secondly, only studies assessing CP and stroke subjects were included. Important information regarding the parameters and underlying measures might have been already studied in other populations and settings that were excluded from the review. It is also possible that we missed additional relevant studies when searching the databases, despite the multiple key words, due to inconsistency in terminology. Furthermore, the included studies were very heterogenic in their methodology, motor tasks, and measures. We categorized the identified measures according to the evaluated parameters. We could not synthesize the findings across the studies to a better extent due to the measures' high variability between the studies. Due to the variability in study design, and the narrative nature of this review we did not assess the methodological quality of the studies. Despite the broad utilization of kinematic analysis in the reviewed studies and despite reports of validity and reliability of some measures, we could not evaluate the appropriateness of measures' selection and the quality of measurements. An additional drawback is that we examined the evidence for the reliability and validity only within the reviewed studies, possibly missing other studies whose sole purpose was to establish these features rather than examine the outcome of a treatment. We suggest that a review with that scope in mind should include additional search of the databases with focus on the reliability, validity, sensitivity, and specificity of the implemented measures, while taking into consideration the variety in the characteristics of studies. This may serve to establish an algorithm for the selection of outcome measures.

## Conclusions

Motor learning is fundamental for improvement of affected motor skills following brain lesion. When designing a rehabilitation training, there is a great importance in selection of appropriate outcome measures for patients' evaluation. In this review, we described the diverse parameters and measures utilized by studies for identification of motor learning in stroke and CP patients.

We overviewed the literature and differentiated the parameters based on the type of motor learning they assess. There is an agreement throughout the studies regarding the meaning and implication of the parameters. However, not all parameters are equally studied. Despite long-lasting effects of practice and generalization of training to real-life movements being a fundamental part of the motor learning theory, the majority of the reviewed studies did not assess the retention and transfer parameters.

Similar metrics can be utilized to measure and quantify different parameters, without evident exclusivity of measures to a specific parameter. Different metrics, the timing and method of their implementation, might reveal inconsistent results regarding a patient's ability to learn. Utilization of solely clinical metrics or the lack of movement quality measurements might only estimate the learning of compensatory movements, rather than of recovery. Additionally, restriction of compensatory movements might result in learning of solely simple movements, with consequences of little generalization to real-life functional movements. The necessity to differentiate between recovery and compensation, and between learning and execution deficiencies, suggests that a combination of measures assessing different features of learning and function might provide a more accurate information about patients' abilities and progress. There is no consensus about the relations between clinical and motor learning measures, and measures described in this study are seldom available in clinical settings. Therefore, we suggest that joint effort by researchers and practitioners should focus on translation of research findings into feasible clinical practices.

To conclude, we have performed a comprehensive data collection of measures used to infer motor learning. While the extant and variability of the measurements produced a raw descriptive review, it should be used as a stepping stone. First, toward qualitative comparison of measurements. Ultimately, we hope it will lead toward an algorithm of outcome measure selection according to population, intervention and concordance with the different motor learning theories, a tool that is currently lacking. Researchers set to examine motor learning should address the entire set of parameters described in motor learning theory, and clinicians should know what measurements are the most valid and reliable to assess treatment progress. Until such an algorithm is developed, intelligible study design and reasoned measurement selection can both improve current studies, and generate the data required to develop the algorithm for future research.

## Author contributions

NS and SB decided on the review's subject, selected the search terms and performed the literature search. NS wrote the review, tables and figures. SB and IM supervised, commended and critically revised the manuscript. All authors substantially contributed to this review and all approved the final version.

## Funding

This work was partially supported by the Helmsley Charitable Trust through the Agricultural, Biological and Cognitive Robotics Initiative of Ben-Gurion University of the Negev, Israel and supported by a Master degree scholarship from faculty of health sciences at Ben-Gurion University of the Negev, Israel.

### Conflict of interest statement

The authors declare that the research was conducted in the absence of any commercial or financial relationships that could be construed as a potential conflict of interest. The reviewer PL and handling Editor declared their shared affiliation, and the handling Editor states that the process nevertheless met the standards of a fair and objective review.

## Abbreviations

AMI: active movement index
ARAT: action research arm test
BAT: bilateral arm training
BBT: box and block test
CP: cerebral palsy
CIMT: constrained induced movement therapy
CT: continuous tracking
CV: coefficient of variation
FMA: fugl-meyer motor assessment
FIM: functional independence measure
ICF: international classification of functioning, disability and health
MD: mean distance
MGA: maximum grip aperture
MS: movement smoothness
MT: movement time
MVT: maximal voluntary torque
NMT: normalized movement time
NMU: normalized number of movement units
nPL: normalized path length
PV: peak velocity
RMSE: root mean square error
RT: reaction time
SRTT: serial reaction time task
%TPV: percentage time to peak velocity
WMFT: wolf motor function test.

## References

[B1] Aluru V. Lu Y. Leung A. Verghese J. Raghavan P. (2014). Effect of auditory constraints on motor performance depends on stage of recovery post-stroke. Front. Neurol. 5:106. 10.3389/fneur.2014.00106 25002859 PMC4066443

[B2] Bastian A. J. (2008). Understanding sensorimotor adaptation and learning for rehabilitation. Curr. Opin. Neurol. 21, 628–633. 10.1097/WCO.0b013e328315a293 18989103 PMC2954436

[B3] Bax M. Goldstein M. Rosenbaum P. Leviton A. Paneth N. Dan B.. (2005). Proposed definition and classification of cerebral palsy, April 2005. Dev. Med. Child Neurol. 47, 571–576. 10.1017/S001216220500112X 16108461

[B4] Bernstein N. (1967). The Co-ordination and Regulation of Movements. Oxford: Pergamon Press.

[B5] Bourke T. C. Coderre A. M. Bagg S. D. Dukelow S. P. Norman K. E. Scott S. H. (2015). Impaired corrective responses to postural perturbations of the arm in individuals with subacute stroke. J. Neuroeng. Rehabil. 12:7. 10.1186/1743-0003-12-7 25605126 PMC4320520

[B6] Boyd L. A. Winstein C. J. (2001). Implicit motor-sequence learning in humans following unilateral stroke: the impact of practice and explicit knowledge. Neurosci. Lett. 298, 65–69. 10.1016/S0304-3940(00)01734-1 11154837

[B7] Boyd L. A. Winstein C. J. (2004). Providing explicit information disrupts implicit motor learning after basal ganglia stroke. Learn. Mem. 11, 388–396. 10.1101/lm.80104 15286181 PMC498316

[B8] Boyd L. A. Winstein C. J. (2006). Explicit information interferes with implicit motor learning of both continuous and discrete movement tasks after Stroke. J. Neurol. Phys. Ther. 30, 46–57. 10.1097/01.npt.0000282566.48050.9b 16796767

[B9] Bruininks R. H. (1978). Bruininks-Oseretsky Test of Motor Proficiency. Circle Pines, MN: American Guidance Service.

[B10] Burtner P. A. Leinwand R. Sullivan K. J. Goh H. T. Kantak S. S. (2014). Motor learning in children with hemiplegic cerebral palsy: feedback effects on skill acquisition. Dev. Med. Child Neurol. 56, 259–266. 10.1111/dmcn.12364 24438099

[B11] Cahill L. McGaugh J. L. Weinberger N. M. (2001). The neurobiology of learning and memory: some reminders to remember. Trends Neurosci. 24, 578–581. 10.1016/S0166-2236(00)01885-3 11576671

[B12] Caimmi M. Carda S. Giovanzana C. Maini E. S. Sabatini A. M. Smania N.. (2008). Using kinematic analysis to evaluate constraint-induced movement therapy in chronic stroke patients. Neurorehabil. Neural Repair 22, 31–39. 10.1177/1545968307302923 17595381

[B13] Cano-de-la-Cuerda R. Molero-Sánchez A. Carratalá-Tejada M. Alguacil-Diego I. M. Molina-Rueda F. Miangolarra-Page J. C. (2015). Theories and control models and motor learning: clinical applications in neurorehabilitation. Neurología 30, 32–41. 10.1016/j.nrleng.2011.12.012 22341985

[B14] Carr J. H. Shepherd R. B. (eds.). (1987). A motor learning model for rehabilitation, in Movement Science Foundations for Physical Therapy in Rehabilitation, 2nd Edn. (Aspen Publishers), 31–91.

[B15] Carroll D. (1965). A quantitative test of upper extremity function. J. Chronic Dis. 18, 479–491. 10.1016/0021-9681(65)90030-5 14293031

[B16] Casadio M. Sanguineti V. (2012). Learning, retention, and slacking: a model of the dynamics of recovery in robot therapy. IEEE Trans. Neural Syst. Rehabil. Eng. 20, 286–296. 10.1109/TNSRE.2012.2190827 22531822

[B17] Chang J. J. Tung W. L. Wu W. L. Huang M. H. Su F. C. (2007). Effects of robot-aided bilateral force-induced isokinetic arm training combined with conventional rehabilitation on arm motor function in patients with chronic stroke. Arch. Phys. Med. Rehabil. 88, 1332–1338. 10.1016/j.apmr.2007.07.016 17908578

[B18] Chen C. L. Kang L. J. Hong W. H. Chen F. C. Chen H. C. Wu C. Y. (2012). Effect of therapist-based constraint-induced therapy at home on motor control, motor performance and daily function in children with cerebral palsy: a randomized controlled study. Clin. Rehabil. 27, 236–245. 10.1177/0269215512455652 22952304

[B19] Chen H. C. Chen C. L. Kang L. J. Wu C. Y. Chen F. C. Hong W. H. (2014). Improvement of upper extremity motor control and function after home-based constraint induced therapy in children with unilateral cerebral palsy: immediate and long-term effects. Arch. Phys. Med. Rehabil. 95, 1423–1432. 10.1016/j.apmr.2014.03.025 24742939

[B20] Christopher S. M. Johnson M. J. (2014). Task-oriented robot-assisted stroke therapy of paretic limb improves control in a unilateral and bilateral functional drink task: a case study. Conf. IEEE Eng. Med. Biol. Soc. 2014, 1194–1197. 10.1109/EMBC.2014.6943810 25570178 PMC6290990

[B21] Cirstea M. C. Levin M. F. (2007). Improvement of arm movement patterns and endpoint control depends on type of feedback during practice in stroke survivors. Neurorehabil. Neural Repair 21, 398–411. 10.1177/1545968306298414 17369514

[B22] Colombo R. Pisano F. Micera S. Mazzone A. Delconte C. Carrozza M. C.. (2008). Assessing mechanisms of recovery during robot-aided neurorehabilitation of the upper limb. Neurorehabil. Neural Repair 22, 50–63. 10.1177/1545968307303401 17626223

[B23] Colombo R. Sterpi I. Mazzone A. Delconte C. Minuco G. Pisano F. (2010). Measuring changes of movement dynamics during robot-aided neurorehabilitation of stroke patients. IEEE Trans. Neural Syst. Rehabil. Eng. 18, 75–85. 10.1109/TNSRE.2009.2028831 19666344

[B24] Dancause N. Ptito A. Levin M. F. (2002). Error correction strategies for motor behavior after unilateral brain damage: short-term motor learning processes. Neuropsychologia 40, 1313–1323. 10.1016/S0028-3932(01)00218-4 11931934

[B25] Dipietro L. Krebs H. I. Fasoli S. E. Volpe B. T. Hogan N. (2009). Submovement changes characterize generalization of motor recovery after stroke. Cortex 45, 318–324. 10.1016/j.cortex.2008.02.008 18640668

[B26] Dipietro L. Krebs H. I. Fasoli S. E. Volpe B. T. Stein J. Bever C.. (2007). Changing motor synergies in chronic stroke. J. Neurophysiol. 98, 757–768. 10.1152/jn.01295.2006 17553941

[B27] Dipietro L. Krebs H. I. Volpe B. T. Stein J. Bever C. Mernoff S. T.. (2012). Learning, not adaptation, characterizes stroke motor recovery: evidence from kinematic changes induced by robot-assisted therapy in trained and untrained task in the same workspace. IEEE Trans. Neural Syst. Rehabil. Eng. 20, 48–57. 10.1109/TNSRE.2011.2175008 22186963 PMC4687974

[B28] Durham K. F. Sackley C. M. Wright C. C. Wing A. M. Edwards M. G. van Vliet P. (2014). Attentional focus of feedback for improving performance of reach-to-grasp after stroke: a randomised crossover study. Physiotherapy 100, 108–115. 10.1016/j.physio.2013.03.004 23796803

[B29] Fitts P. M. (1954). The information capacity of the human motor system in controlling the amplitude of movement. J. Exp. Psychol. 47, 381–391. 10.1037/h0055392 13174710

[B30] Fitts P. M. Posner M. I. (1967). Human Performance. Belmont, CA: Brooks/Cole Pub. Co.

[B31] Forzano L.-A. B. Gravetter F. J. (2009). Research Methods for the Bahavioral Sciences. Belmont, CA: Wadsworth.

[B32] Fugl-Meyer A. R. Jääskö L. Leyman I. Olsson S. Steglind S. (1975). The post-stroke hemiplegic patient. 1. a method for evaluation of physical performance. Scand. J. Rehabil. Med. 7, 13–31. 1135616

[B33] Geerdink Y. Aarts P. Geurts A. C. (2013). Motor learning curve and long-term effectiveness of modified constraint-induced movement therapy in children with unilateral cerebral palsy: a randomized controlled trial. Res. Dev. Disabil. 34, 923–931. 10.1016/j.ridd.2012.11.011 23291509

[B34] Gentile A. M. (1972). A working model of skill acquisition with application to teaching. Quest 17, 3–23. 10.1080/00336297.1972.10519717

[B35] Gilliaux M. Renders A. Dispa D. Holvoet D. Sapin J. Dehez B.. (2015). Upper limb robot-assisted therapy in cerebral palsy: a single-blind randomized controlled trial. Neurorehabil. Neural Repair 29, 183–192. 10.1177/1545968314541172 25015650

[B36] Gladstone D. J. Danells C. J. Black S. E. (2002). The fugl-meyer assessment of motor recovery after stroke: a critical review of its measurement properties. Neurorehabil. Neural Repair 16, 232–240. 10.1177/154596802401105171 12234086

[B37] Goldsmith J. Greven S. Crainiceanu C. (2013). Corrected confidence bands for functional data using principal components. Biometrics 69, 41–51. 10.1111/j.1541-0420.2012.01808.x 23003003 PMC3962763

[B38] Hamilton B. B. Laughlin J. A. Fiedler R. C. Granger C. V. (1994). Interrater reliability of the 7-level functional independence measure (FIM). Scand. J. Rehabil. Med. 26, 115–119. 7801060

[B39] Hemayattalab R. Arabameri E. Pourazar M. Ardakani M. D. Kashefi M. (2013). Effects of self-controlled feedback on learning of a throwing task in children with spastic hemiplegic cerebral palsy. Res. Dev. Disabil. 34, 2884–2889. 10.1016/j.ridd.2013.05.008 23810928

[B40] Hemayattalab R. Rostami L. R. (2010). Effects of frequency of feedback on the learning of motor skill in individuals with cerebral palsy. Res. Dev. Disabil. 31, 212–217. 10.1016/j.ridd.2009.09.002 19864110

[B41] Himmelmann K. (2013). Epidemiology of cerebral palsy. Handb. Clin. Neurol. 111, 163–167. 10.1016/B978-0-444-52891-9.00015-4 23622160

[B42] Huang V. S. Krakauer J. W. (2009). Robotic neurorehabilitation: a computational motor learning perspective. J. Neuroeng. Rehabil. 6:5. 10.1186/1743-0003-6-5 19243614 PMC2653497

[B43] Jongbloed-Pereboom M. Nijhuis-van der Sanden M. W. Steenbergen B. (2013). Norm scores of the box and block test for children ages 3-10 years. Am. J. Occup. Ther. 67, 312–318. 10.5014/ajot.2013.006643 23597689

[B44] Kantak S. S. Winstein C. J. (2012). Learning-performance distinction and memory processes for motor skills: a focused review and perspective. Behav. Brain Res. 228, 219–231. 10.1016/j.bbr.2011.11.028 22142953

[B45] Kelso J. S. (1997). Dynamic Patterns: The Self-Organization of Brain and Behavior. Cambridge, MA: MIT Press.

[B46] Kitago T. Goldsmith J. Harran M. Kane L. Berard J. Huang S.. (2015). Robotic therapy for chronic stroke: general recovery of impairment or improved task-specific skill? J. Neurophysiol. 114, 1885–1894. 10.1152/jn.00336.2015 26180120 PMC4575974

[B47] Kitago T. Krakauer J. W. (2013). Motor learning principles for neurorehabilitation. Handb. Clin. Neurol. 110, 93–103. 10.1016/b978-0-444-52901-5.00008-3 23312633

[B48] Kitago T. Liang J. Huang V. S. Hayes S. Simon P. Tenteromano L.. (2013). Improvement after constraint-induced movement therapy: recovery of normal motor control or task-specific compensation? Neurorehabil. Neural Repair 27, 99–109. 10.1177/1545968312452631 22798152

[B49] Krakauer J. W. (2006). Motor learning: its relevance to stroke recovery and neurorehabilitation. Curr. Opin. Neurol. 19, 84–90. 10.1097/01.wco.0000200544.29915.cc 16415682

[B50] Krakauer J. W. Mazzoni P. (2011). Human sensorimotor learning: adaptation, skill, and beyond. Curr. Opin. Neurobiol. 21, 636–644. 10.1016/j.conb.2011.06.012 21764294

[B51] Krebs H. I. Fasoli S. E. Dipietro L. Fragala-Pinkham M. Hughes R. Stein J.. (2012). Motor learning characterizes habilitation of children with hemiplegic cerebral palsy. Neurorehabil. Neural Repair 26, 855–860. 10.1177/1545968311433427 22331211 PMC4688005

[B52] Krebs H. I. Hogan N. Hening W. Adamovich S. V. Poizner H. (2001). Procedural motor learning in Parkinson's disease. Exp. Brain Res. 141, 425–437. 10.1007/s002210100871 11810137

[B53] Lai S. M. Studenski S. Duncan P. W. Perera S. (2002). Persisting consequences of stroke measured by the stroke impact scale. Stroke 33, 1840–1844. 10.1161/01.STR.0000019289.15440.F2 12105363

[B54] Levin M. F. Kleim J. A. Wolf S. L. (2009). What do motor “recovery” and “compensation” mean in patients following stroke? Neurorehabil. Neural Repair 23, 313–319. 10.1177/1545968308328727 19118128

[B55] Lloyd-Jones D. Adams R. J. Brown T. M. Carnethon M. Dai S. De Simone G.. (2010). Executive summary: heart disease and stroke statistics-2010 update: a report from the american heart association. Circulation 121, 46–215. 10.1161/CIRCULATIONAHA.109.192667 20177011

[B56] Lyle R. C. (1981). A performance test for assessment of upper limb function in physical rehabilitation treatment and research. Int. J. Rehabil. Res. 4, 483–492. 7333761 10.1097/00004356-198112000-00001

[B57] Masia L. Frascarelli F. Morasso P. Di Rosa G. Petrarca M. Castelli E.. (2011). Reduced short term adaptation to robot generated dynamic environment in children affected by Cerebral Palsy. J. Neuroeng. Rehabil. 8:28. 10.1186/1743-0003-8-28 21600031 PMC3117777

[B58] Massie C. Malcolm M. P. Greene D. Thaut M. (2009). The effects of constraint-induced therapy on kinematic outcomes and compensatory movement patterns: an exploratory study. Arch. Phys. Med. Rehabil. 90, 571–579. 10.1016/j.apmr.2008.09.574 19345771

[B59] Mawase F. Bar-Haim S. Karniel A. (2011). Lack of predictive control in lifting series of virtual objects by individuals with diplegic cerebral palsy. IEEE Trans. Neural Syst. Rehabil. Eng. 19, 686–695. 10.1109/TNSRE.2011.2170589 21984525

[B60] Molier B. I. Prange G. B. Krabben T. Stienen A. H. van der Kooij H. Buurke J. H.. (2011). Effect of position feedback during task-oriented upper-limb training after stroke: five-case pilot study. J. Rehabil. Res. Dev. 48, 1109–1117. 10.1682/JRRD.2010.07.0128 22234715

[B61] Nichols-Larsen D. S. Clark P. C. Zeringue A. Greenspan A. Blanton S. (2005). Factors influencing stroke survivors' quality of life during subacute recovery. Stroke 36, 1480–1484. 10.1161/01.STR.0000170706.13595.4f 15947263

[B62] Nourrit-Lucas D. Zelic G. Deschamps T. Hilpron M. Delignières D. (2013). Persistent coordination patterns in a complex task after 10 years delay. Subtitle: how validate the old saying “Once you have learned how to ride a bicycle, you never forget!” Hum. Mov. Sci. 32, 1365–1378. 10.1016/j.humov.2013.07.005 24054437

[B63] Orrell A. J. Eves F. F. Masters R. S. MacMahon K. M. M. (2007). Implicit sequence learning processes after unilateral stroke. Neuropsychol. Rehabil. 17, 335–354. 10.1080/09602010600832788 17474060

[B64] Patton J. L. Stoykov M. E. Kovic M. Mussa-Ivaldi F. A. (2006). Evaluation of robotic training forces that either enhance or reduce error in chronic hemiparetic stroke survivors. Exp. Brain Res. 168, 368–383. 10.1007/s00221-005-0097-8 16249912

[B65] Pohl P. S. McDowd J. M. Filion D. Richards L. G. Stiers W. (2006). Implicit learning of a motor skill after mild and moderate stroke. Clin. Rehabil. 20, 246–253. 10.1191/0269215506cr916oa 16634344

[B66] Raghavan P. Krakauer J. W. Gordon A. M. (2006). Impaired anticipatory control of fingertip forces in patients with a pure motor or sensorimotor lacunar syndrome. Brain 129, 1415–1425. 10.1093/brain/awl070 16597653 PMC2093998

[B67] Rohrer B. Fasoli S. Krebs H. I. Volpe B. Frontera W. R. Stein J.. (2004). Submovements grow larger, fewer, and more blended during stroke recovery. Motor Control 8, 472–483. 10.1123/mcj.8.4.472 15585902

[B68] Sainburg R. L. Wang J. (2002). Interlimb transfer of visuomotor rotations: independence of direction and final position information. Exp. Brain Res. 145, 437–447. 10.1007/s00221-002-1140-7 12172655 PMC10704413

[B69] Schaefer S. Y. Patterson C. B. Lang C. E. (2013). Transfer of training between distinct motor tasks after stroke: implications for task-specific approaches to upper-extremity neurorehabilitation. Neurorehabil. Neural Repair 27, 602–612. 10.1177/1545968313481279 23549521 PMC3769167

[B70] Scheidt R. A. Stoeckmann T. (2007). Reach adaptation and final position control amid environmental uncertainty after stroke. J. Neurophysiol. 97, 2824–2836. 10.1152/jn.00870.2006 17267755

[B71] Schmidt R. A. (1988). Motor Control and Learning: A Behavioral Emphasis, 2nd Edn. Champaign, IL: Human Kinetics.

[B72] Schmidt R. A. Lee T. D. (2004). Motor control and learning: A Behavioral Emphasism, 4th Edn. Champaign, IL: Human Kinetics.

[B73] Schmidt R. A. Wrisberg C. A. (2008). Motor Learning and Performance: A Situation-Based Learning Approach, 4th Edn. Champaign, IL: Human Kinetics.

[B74] Senesac C. R. Davis S. Richards L. (2010). Generalization of a modified form of repetitive rhythmic bilateral training in stroke. Hum. Mov. Sci. 29, 137–148. 10.1016/j.humov.2009.05.004 19892424

[B75] Shadmehr R. (2004). Generalization as a behavioral window to the neural mechanisms of learning internal models. Hum. Mov. Sci. 23, 543–568. 10.1016/j.humov.2004.04.003 15589621 PMC2722915

[B76] Sivan M. O'Connor R. J. Makower S. Levesley M. Bhakta B. (2011). Systematic review of outcome measures used in the evaluation of robot-assisted upper limb exercise in stroke. J. Rehabil. Med. 43, 181–189. 10.2340/16501977-0674 21305232

[B77] Sterpi I. Panarese A. Micera S. Pisano F. Colombo R. (2012). The generalization of motor recovery after stroke: assessment within and outside the training workspace in 2012 4th IEEE RAS & EMBS International Conference on Biomedical Robotics and Biomechatronics (BIOROB) (Rome: IEEE Press), 1022–1025.

[B78] Subramanian S. K. Yamanaka J. Chilingaryan G. Levin M. F. (2010). Validity of movement pattern kinematics as measures of arm motor impairment poststroke. Stroke 41, 2303–2308. 10.1161/STROKEAHA.110.593368 20814001

[B79] Takahashi C. D. Reinkensmeyer D. J. (2003). Hemiparetic stroke impairs anticipatory control of arm movement. Exp. Brain Res. 149, 131–140. 10.1007/s00221-002-1340-1 12610680

[B80] Tanaka S. Sandrini M. Cohen L. G. (2011). Modulation of motor learning and memory formation by non-invasive cortical stimulation of the primary motor cortex. Neuropsychol. Rehabil. 21, 650–675. 10.1080/09602011.2011.605589 21942897

[B81] Thaut M. H. Kenyon G. P. Hurt C. P. McIntosh G. C. Hoemberg V. (2002). Kinematic optimization of spatiotemporal patterns in paretic arm training with stroke patients. Neuropsychologia 40, 1073–1081. 10.1016/S0028-3932(01)00141-5 11900758

[B82] Wiklund L. M. Uvebrant P. (1991). Hemiplegic cerebral palsy: correlation between CT morphology and clinical findings. Dev. Med. Child Neurol. 33, 512–523. 10.1111/j.1469-8749.1991.tb14916.x 1864477

[B83] Wolf S. L. Lecraw D. E. Barton L. A. Jann B. B. (1989). Forced use of hemiplegic upper extremities to reverse the effect of learned nonuse among chronic stroke and head-injured patients. Exp. Neurol. 104, 125–132. 10.1016/S0014-4886(89)80005-6 2707361

[B84] Wu C. Y. Chen C. L. Tang S. F. Lin K. C. Huang Y. Y. (2007). Kinematic and clinical analyses of upper-extremity movements after constraint-induced movement therapy in patients with stroke: a randomized controlled trial. Arch. Phys. Med. Rehabil. 88, 964–970. 10.1016/j.apmr.2007.05.012 17678656

[B85] WHO (2006). Neurological Disorders: Public Health Challenges. 41–176.

[B86] Wu C. Y. Chuang L. L. Lin K. C. Chen H. C. Tsay P. K. (2011). Randomized trial of distributed constraint-induced therapy versus bilateral arm training for the rehabilitation of upper-limb motor control and function after stroke. Neurorehabil. Neural Repair 25, 130–139. 10.1177/1545968310380686 20947493

[B87] Yao F. Müller H. G. Wang J. L. (2005). Functional data analysis for sparse longitudinal data. J. Am. Stat. Assoc. 100, 577–590. 10.1198/016214504000001745

